# Folfiri-Aflibercept vs. Folfiri-Bevacizumab as Second Line Treatment of RAS Mutated Metastatic Colorectal Cancer in Real Practice

**DOI:** 10.3389/fonc.2019.00766

**Published:** 2019-08-13

**Authors:** Alessandro Ottaiano, Monica Capozzi, Salvatore Tafuto, Alfonso De Stefano, Chiara De Divitiis, Carmela Romano, Antonio Avallone, Guglielmo Nasti

**Affiliations:** ^1^SSD Innovative Therapy for Abdominal Metastases, Naples, Italy; ^2^Clinical and Experimental Abdominal Oncology of the National Cancer Institute of Naples, Istituto Nazionale Tumori di Napoli, IRCCS “G. Pascale”, Naples, Italy

**Keywords:** colorectal cancer, chemotherapy, bevacizumab, aflibercept, real practice

## Abstract

**Background:** There are no clinical studies comparing the efficacy of bevacizumab vs.aflibercept in association with folfiri in RAS mutated (RAS-M) metastatic colorectal cancer patients (mCRC) pretreated with folfox and bevacizumab.

**Patients and Methods:** Consecutive RAS-M unresectable mCRC patients progressing to first-line folfox/bevacizumab were treated with 12 cycles of folfiri/bevacizumab (arm A) or folfiri/aflibercept (arm B) at Oncologist discretion. Differences in overall survival between the two schedules were analyzed. Responses and toxicities were described with RECIST and NCI-CTC v4.0, respectively.

**Results:** Seventy-four patients were treated from January 2014 to January 2018; 31 with arm A, 43 with arm B. Among clinical factors there was a predominance of more extended disease (>2 metastatic sites) in arm B (26/43 [60.5%] vs. 10/31 [32.2%] arm A; *p* = 0.0414). Fifty-nine patients were evaluable for response: arm A, 5 PR (Partial Response), 15 SD (Stable Disease), 8 PD (Progressive Disease); arm B, 5 PR, 16 SD, 10 PD. There were no grade 4 toxic events. Duration of first-line chemotherapy was significantly shorter in patients treated in arm B (12 pts <6 months, 16 pts ≥6, and <12, 15 pts ≥12) vs. arm A (1 pts <6 months, 14 pts ≥6, and <12, 16 pts ≥12) (*p* = 0.0210); these patients were excluded from survival analysis to avoid prognostic interferences. No maintenance treatment with aflibercept was done in arm B while in arm A bevacizumab with or without fluorouracil and folinic acid were allowed. Median OS were 8.9 months in arm A vs. 12.1 months in arm B (+3.2 months; *p* = 0.9331, HR: 1.02; 95% CI: 0.57–1.84). Six-months survivals were 65% in arm A and 80% in arm B.

**Conclusions:** Folfiri/bevacizumab and folfiri/aflibercept are equally effective second-line therapies in RAS-M mCRC patients. Although not significant, folfiri/aflibercept was associated with a lower risk of death particularly during the 6-months induction phase.

## Introduction

Colorectal cancer (CRC) is a leading cause of cancer-related deaths worldwide. About 30% of patients presents with metastatic disease at diagnosis and half of patients with localized disease will develop it later after surgery (1).

The treatment of metastatic colorectal cancer (mCRC) relies on the administration of chemotherapies (fluoropyrimidines, oxaliplatin and irinotecan) and biologics (bevacizumab, cetuximab, panitumumab, aflibercept, regorafenib) (2). Regorafenib is a new generation multi-tyrosine-kinase inhibitor orally administered as single agent at failure of the above cited therapies (3). Anti-EGFR (Epidermal Growth Factor Receptors) drugs (cetuximab and panitumumab) are monoclonal antibodies administered both as single agents or with chemotherapy in first, second or third-line setting of mCRC; they bind to EGFR preventing the activation of downstream signal proteins involved in promoting proliferation of neoplastic cells (4, 5). However, their use is recommended only in mCRC patients with wild-type K- and N-RAS (Kirsten and Neuroblastoma RAt Sarcoma) oncogenes because their mutations lead to constitutive activation of the EGFR pathway and many studies demonstrated their strong predictive power (6–8). Bevacizumab is a monoclonal antibody targeting the vascular endothelial growth factor (VEGF) and preventing its angiogenic effects; nowadays, bevacizumab is indicated in association with chemotherapy in both first- and second-lines chemotherapies of mCRC patients (9, 10). Clinical studies have demonstrated that chemotherapy doublets (fluorouracil and oxaliplatin or fluorouracil and irinotecan, briefly indicated as folfox and folfiri) in association with bevacizumab are active independently from RAS status (11, 12) and that the continuation of bevacizumab with second-line chemotherapy beyond progression is associated with improved survival compared to chemotherapy alone (13, 14).

Aflibercept, a recombinant fusion protein between the Fc portion of IgG1 and binding portions of VEGFR 1 and 2, is an anti-angiogenic agent targeting both VEGFA and PlGF (Placental Growth Factor) (15). A pivotal randomized study demonstrated that addition of aflibercept to folfiri after progression to an oxalipltain-based first-line chemotherapy improves survival (16) and a recent *post-hoc* analysis has shown that efficacy of second-line treatment with folfiri/aflibercept is not dependent from the RAS status (17). Thus, folfiri/aflibercept is a further suitable option in second-line treatment of RAS-M mCRC patients.

To date there are no randomized studies to drive the selection of the best anti-angiogenic drug (bevacizumab beyond progression vs. aflibercept) after failure of the first-line chemotherapy in RAS-M mCRC patients.

The present study is the first one reporting results on folfiri/bevacizumab vs. folfiri/aflibercept in KRAS-M mCRC patients progressing at first-line folfox/bevacizumab in a non-randomized real practice cohort.

## Patients and Methods

### Study Design and Sources of Data

**Figure 1:**
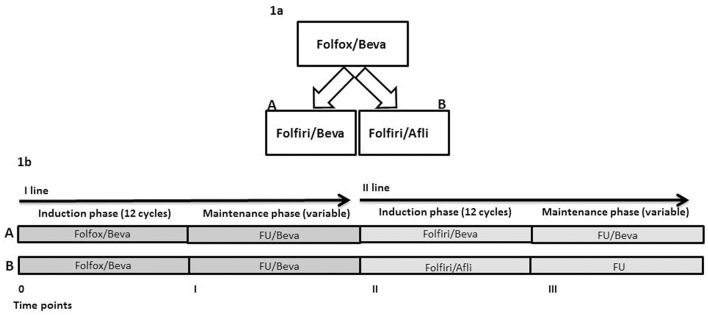
**(A)** Study design. **(B)** Schematic representation of study time points.

This is a real practice, retrospective, non-randomized study. The sources of data were the clinical records and the AIFA (*Agenzia Italiana del Farmaco*, italian governative agency for pharmaceutical products) registry. KRAS-M unresectable mCRC patients were treated at the oncologist discretion at progression to first-line folfox/bevacizumab with folfiri/bevaciaumb (arm A) or folfiri/aflibercept (Afli) (arm B) (Figure 1A). These are two standard and registered suitable options in the second-line treatment of KRAS-M mCRC patients for ESMO (18) and NCCN (NCCN guidelines v3.2018). No internal/institutional guidelines were available nor applied for the second-line choice. The criteria for patients' treatment were: age <80 years, life expectancy of at least 3 months, PS ECOG <3, adequate renal, liver and cardiac functions. The primary outcome of this study was the description of overall survival (OS), secondary objectives were description of activity and toxicity.

### Definition of “Unresectable” Disease

All patients of the present cohort were unresectable at the start of second-line chemotherapy. The resection of metastases was judged in a multidisciplinary context involving the Oncologists, the Surgeons and the Radiologists. Patients were considered unresectable if they presented lesion/s not removable with R0 margins and/or not appropriate residual organ/s function (liver and/or lung).

### Drugs and Administration Schedules

Patients were treated at the Department of Abdominal Medical Oncology of the National Cancer Institute (Naples, Italy) from October 2014 to September 2017. Written informed consent was obtained from all patients before starting therapy. All primary tumors were KRAS-M. All patients received first-line chemotherapy based on mFOLFOX6 (briefly defined as folfox) and bevacizumab regimen (oxaliplatin 85 mg/m^2^, l-leucovorin 200 mg/m^2^, bolus 5-FU 400 mg/m^2^, 46-h infusion of 5-FU 2,400 mg/m^2^, bevacizumab 5 mg/kg on day 1 of each 14-day cycle) up to 12 cycles, then they received maintenance treatment with 5-FU and bevacizumab (FU/Bevacizumab: l-leucovorin 200 mg/m^2^, bolus 5-FU 400 mg/m^2^, 46-h infusion of 5-FU 2,400 mg/m^2^, bevacizumab 5 mg/kg on day 1 of each 14-day cycle) until progression or unacceptable toxicity. Thereafter, 12 cycles of folfiri (irinotecan 180 mg m^2^, l-leucovorin 200 mg/m^2^, bolus 5-FU 400 mg/m^2^, 46-h infusion of 5-FU 2,400 mg/m^2^) with bevacizumab 5 mg/kg (Arm A) or aflibercept 4 mg/kg (Arm B) on day 1 of each 14-day cycle were administered. Patients with responding or stable diseases received maintenance therapy with FU/bevacizumab in Arm A or FU (l-leucovorin 200 mg/m^2^, bolus 5-FU 400 mg/m^2^, 46-h infusion of 5-FU 2,400 mg/m^2^) in Arm B until progression or unacceptable toxicity. Standard premedication with cortisone and antiemetics was applied in both arms. Irinotecan was premedicated also with atropine. Maintenance therapy with chemotherapy (FU or FOLFIRI) and aflibercept was not permitted by our local Pharmacy Authorities considering that in the folfiri/aflibercept arm of the pivotal trial (i) patients received a median of seven administrations of aflibercept and that (ii) in case of chemotherapy discontinuation for any reasons, it was stopped. Folfiri/bevacizumab or folfiri/aflibercept are two suitable options for unresectable KRAS-M mCRC patients as second-line therapy. The choice of second-line chemotherapy regimen (Arm A or Arm B) was based predominantly on Oncologists' preferences. Overall, there were four time points in both arms: (1) time 0, start of first-line chemotherapy (12 cycles of folfox/bevacizumab), (2) time I, start of maintenance therapy (a variable period of FU/bevacizumab), (3) time II, start of second-line chemotherapy (12 cycles of folfiri/bevacizumab in Arm A or folfiri/aflibercept in Arm B), (4) time III, start of maintenance treatment after second-line chemotherapy with a variable period of FU/bevacizumab in arm A and FU in arm B (Figure 1B).

### Patients Management

The response to chemotherapy was evaluated by RECIST (Response Evaluation Criteria In Solid Tumors). Total body computed tomography (CT) scan and CEA (CarcinoEmbryonic Antigen) monitoring were done every 3 months. Complete response (CR) was defined as complete disappearance of all detectable evidence of disease on total body computed tomography. Partial response (PR) was defined as at least a 30% decrease in the sum of diameters of target lesions. Stable disease (SD) was defined as everything between 30% decrease and 20% growth of tumor size. Progressive disease (PD) was defined as at least a 20% increase in the sum of diameters of target lesions. Toxicity was graded with the Common Toxicity Criteria for Adverse Events (CTCAE) v4.0. Treatment drugs modifications or delays were applied in case of toxicity according to Summary of Product Characteristics (SmPC).

### Statistical Analyses and Data Presentation

Results of this study are necessarily descriptive and exploratory. Associations between chemotherapy arms and clinical and pathologic variables were evaluated by χ^2^-test. *P* < 0.05 was considered statistically significant. The primary outcome measure was the Overall Survival (OS). It was measured from the start of the second-line until death from any cause. Progression-free survival was not selected as a study objective because, in most cases, the radiologic monitoring after the induction phase of chemotherapy was conducted outside our Institute. The vital status was the simplest, most solid and reliable outcome to report and analyze. The Kaplan-Meier product limit method was applied to graph OS. Statistical analysis was performed using the MedCalc® 9.3.7.0 and Excel software.

## Results

### Patients' Characteristics

Seventy-four patients affected by KRAS-M unresectable mCRC were treated with first-line chemotherapy based on folfox/bevacizumab. Thereafter, at progression, 31 patients were treated with folfiri/bevacizumab (Arm A), 43 with folfiri/aflibercept (Arm B). Patients' characteristics are displayed in Table 1 according to treatment arms and clinical and pathological characteristics. Median ages were almost similar in both arms. Female were predominant in Arm B (55.8 vs. 45.1% in Arm A) but gender differences were not statistically significant. ECOG Performance Status at start of second-line therapy was quite homogeneously distributed among arms with a predominance of PS 1 (51.6% in arm A, 64.7% in arm B), only two patients in arm A had PS ECOG 2, three in arm B. There were no significant differences regarding the distribution of the primary tumor site. Noteworthy, there was a significant difference between arms in terms of number of organs involved; in fact, in arm B 60.5% of patients presented with more than two metastatic sites compared to 32.2% in arm A (*p* = 0.0414). Previous adjuvant oxaliplatin-based chemotherapy was performed in 41.9% of patients in arm A and 46.5% in arm B. Time-on-first line folfox/bevacizumab was categorized in three classes: <6 months, >6 months, and <12 months, >12 months. All patients interrupted first-line therapy for progression. Median duration of first-line chemotherapies were 13 months in arm A and 8 months in arm B. Interestingly, there was a strong unbalance of patients rapidly progressing to first-line folfox/bevacizumab in Arm B (27.9%) compared to 2.3% in Arm A (*p* = 0.0210). Fifty-nine patients were evaluable for response assessment; the response rate was 16.1% in arm A and 14.7% in arm B, there were no complete responses. Eighty-three percent of patients in Arm A underwent to third-line therapies, 69.7% in Arm B; regorafenib was the most common third-line treatment in this cohort of patients.

**Table 1:** Characteristics of patients and disease according to second-line chemotherapies (Arm A: folfiri/bevacizumab; Arm B: folfiri/aflibercept).

**Characteristics**	**Arm A**	**Arm B**	***P***
**Age, years**			
Median	65	66	
Range	45–76	31–78	0.3429
**Gender**			
Male	17	19	
Female	14	24	0.3689
Performance status^*^			
0	13	18	
1	16	22	
2	2	3	0.9960
**Site of primary tumor**			
Right colon	14	23	
Left colon	17	20	0.4826
Site of metastases^*^			
One	6	3	
Two	15	14	
More than two	10	26	0.0414
**Previous adjuvant chemotherapy**			
Yes	13	20	
No	18	23	0.6980
**Previous surgery of metastatic disease**			
Yes	12	10	
No	19	33	0.1541
**Time on I line chemotherapy**			
<6 months	1	12	
>6 months <12 months	14	16	
>12 months	16	15	0.0210
**Response to II line chemotherapy**			
Complete response	0	0	
Partial response	5	5	
Stable disease	15	16	
Progressive disease	8	10	0.9502
**Type of III line therapies**			
Regorafenib	10	14	
Raltitrexed	3	6	
FOLFOX rechallenge	9	8	
Raltitrexed plus mitomyicin C	4	2	
None	5	13	0.3913

* *At start of II line chemotherapy*.

### Safety and Treatment Exposure

The most frequent G1/G2 toxicities included asthenia, nausea, neutropenia and anemia in both arms. The most common G3 adverse event was neutropenia (4 patients in arm A and 7 in arm B). Cardiovascular G3 events occurred in 2 patients in Arm A and 3 in arm B. All G3 toxicities occurred during the induction phase in both arms. No G4 events were observed. A detailed list *per* patient of hematologic and non-hematologic toxicities is reported in Table 2. Patients older than 75 years or with PS 2 started FOLFIRI at 75% of the planned doses. Overall, chemotherapy was reduced in 32.3% of patients in arm A and 39.5% in arm B. Folfiri dose reductions did not significantly differ among arms (Table 3). In both arms, most of the dose reductions were caused by hematological toxicity (22.9% of total patients, 19.3% arm A vs. 25.6% in arm B), while 12.2 % were due to non-hematological toxicities (12.9% arm A vs. 11.6% in arm B). Although not significant, G3 hematologic toxicities were more frequent in arm B. Chemotherapy delays of more than 2 weeks were documented in 18 patients (24.3 %), with 6 in arm A and 12 in arm B. Such delays occurred at a similar rate in the first four cycles (3 arm A vs. 3 arm B), while after cycle 5, were documented considerably more often in arm B patients (3 arm A vs. 9 arm B) (data not shown). There were no significant differences in hypertension occurrence between patients receiving bevacizumab or aflibercept. Dose reduction of bevacizumab was applied in two patients because of G3 epistaxis, and of aflibercept in one patient because of G3 epistaxis and hypertension. The median time-on-second-line therapy was similar in both arms: 5 months Arm A (95% CI: 4.0–6.2 months) and 5 months arm B (95% CI: 4.0–5.3 months). The median time-on-maintenance therapy was 5.2 months for Arm A (95% CI: 3–6.7) and 4 months for Arm B (95% CI: 2.6–4.0) (see also Figure S1 and Table S1).

**Table 2:** Incidence of maximum grade of adverse event *per* patient according to second-line chemotherapies.

	**Arm A**		**Arm B**	
	**G1/G2**	**G3**	**G1/G2**	**G3**
Neutropenia	11	4	13	7
Anemia	10	2	14	4
Thrombocytopenia	3	–	4	–
Nausea	11	–	13	2
AST/ALT increase	4	–	6	–
Diarrhea	6	2	4	1
Asthenia	12	–	9	3
Hypertension	8	–	10	2
Mucositis	4	1	2	–
Epistaxis	–	2	–	1
Thromboembolic events	1	–	–	–
Blood bilirubin increased	3	1	3	–
Acute kidney injury	–	–	1	–
Dispnea	–	–	2	–

**Table 3:** Comparison of hematologic vs. non-hematologic toxicities and reasons for chemotherapy dose reductions according to second-line chemotherapies.

		**Arm A No**		**Arm B No**		***P***
**Toxicity**		**G1/G2**	**G3**	**G1/G2**	**G3**	
Hematologic		24	6	27	11	0.40
Non-hematogologic		49	6	50	9	0.49
	**Total 74 pts No (%)**	**Arm A 31 pts No (%)**		**Arm B 43 pts No (%)**		
**Reasons for dose treatment reduction**						
Hematologic toxicity	17 (22.9)	6 (19.3)		11 (25.6)		
Non-hematologic toxicity	9 (12.2)	4 (12.9)		5 (11.6)		
Patient request	1 (1.3)	0 (0.0)		1 (2.3)		
No dose reduction	47 (63.5)	21 (67.7)		26 (60.5)		0.97

### Time-To-Outcome Analysis

**Figure 2:**
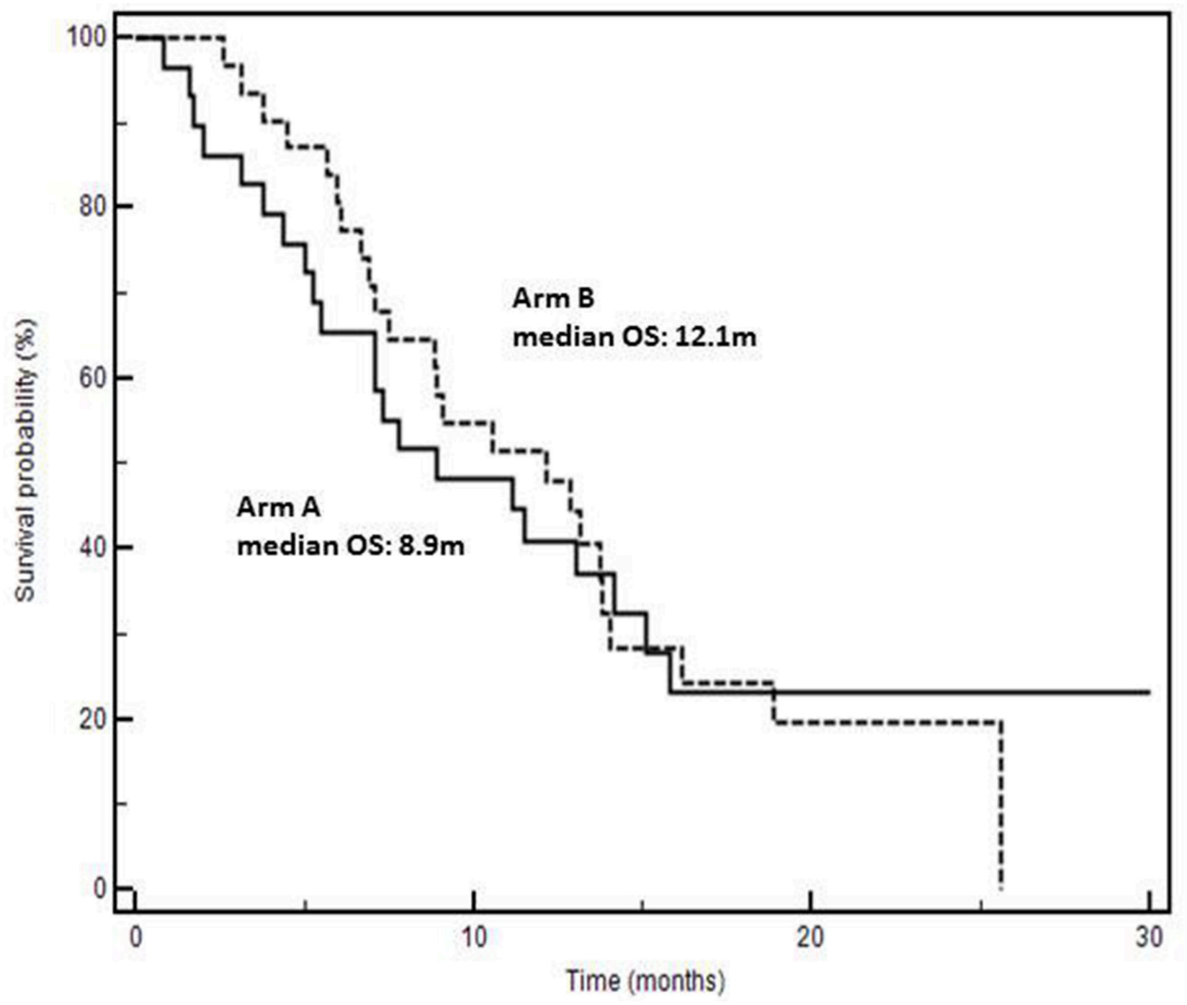
Kaplan-Meyer survival curves according to second-line chemotherapy (Arm A: folfiri/bevacizumab; Arm B: folfiri/aflibercept).

Analysis of OS was done excluding 13 rapidly progressing patients (Table 1) to avoid a clear and strong prognostic interference in favor of Arm A. Median OS were 8.9 in arm A vs. 12.1 months in arm B (+3.2 months; *P* = 0.9331, HR: 1.02; 95% CI: 0.57–1.84) (Figure 2). Details on patients progressing during the induction phase and survival of the entire series are reported in Figure S2 and Table S1.

## Discussion

In the present study we report results of a comparison between two different second-line therapies (folfiri/bevacizumab vs. folfiri/aflibercept) in advanced KRAS-M folfox/bevacizumab-pretreated mCRC patients in a “real world” cohort registered into a national registry (AIFA registry). To date, there are no studies comparing the effect of aflibercept and bevacizumab in this clinical setting.

Three important biases were present: (1) the predominance of more extended disease (> two metastatic sites) in arm B, (2) the duration of first-line chemotherapy which was significantly shorter in patients of arm B, and (3) the lack of a maintenance treatment with aflibercept in arm B. In fact, in arm A, after an induction phase of 6 months (12 cycles), maintenance with bevacizumab was applied while in arm B maintenance with aflibercept was not permitted. The second bias was necessarily “corrected” by excluding 13 (12 of whom were in arm B) rapidly progressing patients from the analysis of time-to-outcome. Surprisingly, although the presence of patients with more advanced disease in Arm B, we found a non-significant early divergence of survival curves in favor of that arm indicating a lower risk of death (Figure 2). The absence of statistical significance is certainly conditioned by the low number of cases and hypothetically by the absence of a biologic maintenance treatment in arm B. In fact, 6-months-survival was 80% in arm B and 65% in arm A; afterwards, the curves overlap gradually. However, these biases do not stultify even if they reinforce the hypothetical positive impact of folfiri/aflibercept in second-line KRAS-M mCRC patients. Real practice studies, often retrospective and exploratory in their nature as the present one, may have a strong hypothesis-generating role. Although the strengths of our study reside on single-center, consecutive and real life scenarios, we cannot rule out the hypothesis that the results could be influenced by uncontrolled and unknown biases due to its non-randomized, retrospective, and small size nature. However, although limited by the size and the non-randomized and retrospective nature, this study may generate an interesting clinical hypothesis: at least theoretically, the higher inhibition of other pro-angiogenic factors than bevacizumab could account for better second-line results. In fact, many evidences show that aflibercept binds to VEGF-A with higher affinity and a faster association rate than bevacizumab, and that it has the additional property to bind VEGF-B and PlGF which have been largely implicated in promoting tumor growth and spread (19, 20). These additional targets may be important in promoting tumor progression in bevacizumab-pretreated mCRC (21) and increasing levels of VEGF-B and PlGF may be implicated in resistance to bevacizumab (22–24). Interestingly, doubling bevacizumab dosage beyond progression does not overcome resistance and not ameliorate clinical results (25). In the present study VEGFs and PlGF levels before starting second-line therapies were not measured; this was a further limit due to its retrospective nature. However, a hypothesis is that aflibercept binds to VEGFA with a higher affinity than bevacizumab and contributes to block the biological effects of both VEGFA and PlGF increase after progression to folfox/bevacizumab. The study of different biologics efficacy in association with chemotherapy will gain increasing importance because, recently, the definition of RAS status has been extended to N-RAS and other biomarkers are enriching the molecular predictive and prognostic scenario of mCRC (i.e., BRAF, PI3K, MET, MSI, etc.) (7, 8). Interestingly, a recent study reported a trend (*p* = 0.08) for better survival in 482 BRAF mutated mCRC patients (+2.7 months) treated with folfiri/aflibercept vs. folfiri/placebo (17).

In summary, we present a hypothesis-generating article on the sequential role of chemotherapy and anti-angiogenic biologics in mCRC patients. We show in line with previously published studies that both aflibercept and bevacizumab are two suitable anti-angiogenic options in the second-line treatment of KRAS mutated mCRC.

## Ethics Statement

This is a real practice, retrospective study conducted according to standard therapeutic strategies. No internal ethics committee approval was required. However, informed consent from each patient was sought.

## Author Contributions

AO contributed in patients' management, planning, analysis, discussing, and writing of the manuscript. MC and AD contributed in planning, analysis, discussing, and writing of the manuscript. ST and CR contributed in patients' management and writing of the manuscript. CD contributed in planning and writing of the manuscript. AA contributed in planning, writing, and discussing of the manuscript. GN contributed in patients' management, planning, writing, and discussing of the manuscript.

### Conflict of Interest Statement

The authors declare that the research was conducted in the absence of any commercial or financial relationships that could be construed as a potential conflict of interest.
