# Supplementary material for: Folfiri-Aflibercept vs. Folfiri-Bevacizumab as Second Line Treatment of RAS Mutated Metastatic Colorectal Cancer in Real Practice
Source: Front Oncol. 2019 Aug 13;9:766. doi: 10.3389/fonc.2019.00766 (PMC6700318; doi:10.3389/fonc.2019.00766)
Supplement: Supplementary file 1 [file Data_Sheet_1.docx]

**SUPPLEMENTARY FILE**

**Figure S1.** Kaplan-Meier curves of treatment continuation probability according to Arm A (Folfiri/Bevacizumb followed by FU/Bevacizumab) and Arm B (Folfiri/Aflibercept followed by FU).


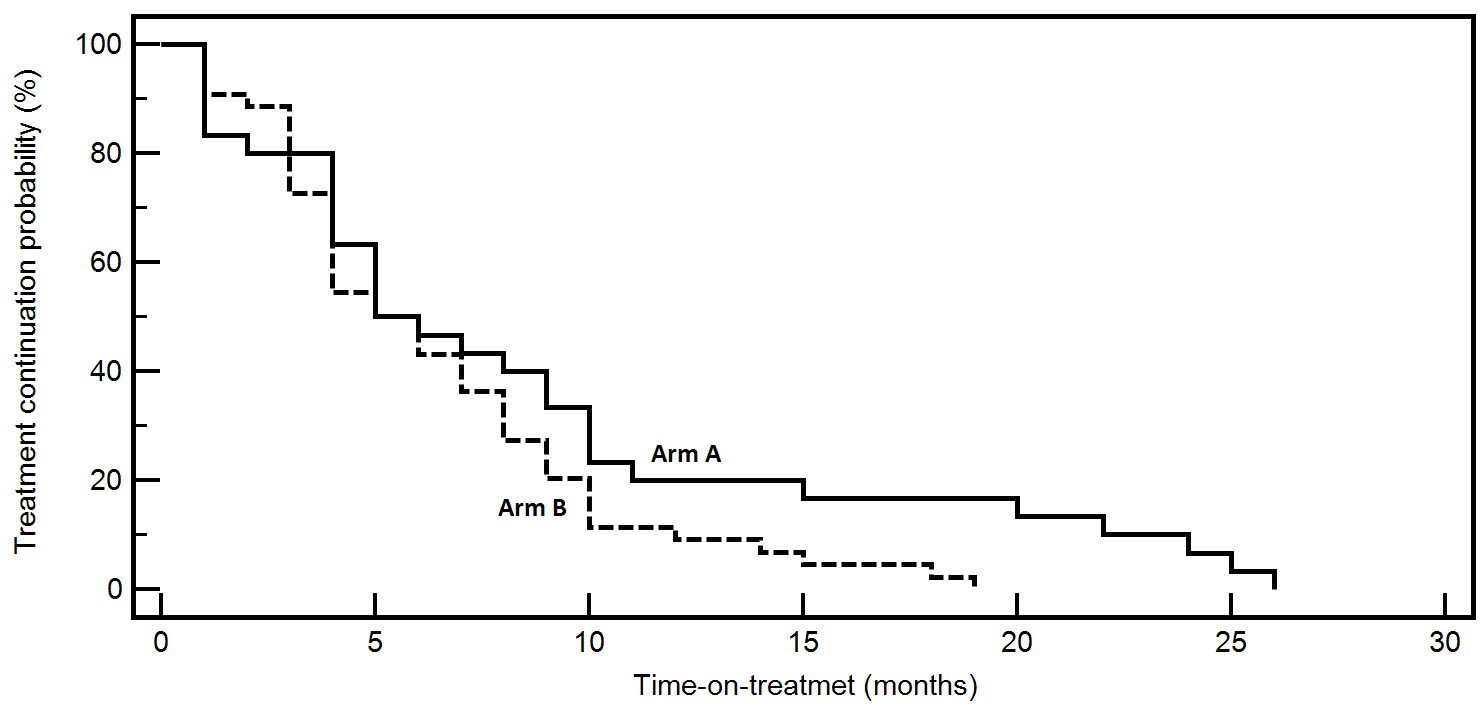


**Table S1.** Section I. Duration of treatments in the whole series according to I and II therapy lines. Section II. Number of treated patient according to I and II therapy lines; number of progressive diseases registered during the induction phases.

|  | **I line treatment**  **(Folfox/Beva)** | **II line Arm A**  **(Folfiri/Beva)** | **M-Arm A**  **(FU/Beva)^1^** | **II line Arm B**  **(Folfiri/Afli)** | **M-Arm B**  **(FU)^1^** |
| --- | --- | --- | --- | --- | --- |
| ***Section I***    **Duration of treatment**  **(months)** |  |  |  |  |  |
| **Median** | Whole: 10.5  Arm A: 13.0;  Arm B: 8.0 | 5.0 | 5.2 | 5.0 | 4.0 |
| **Ranges (lowest-highest)** | Whole: 3.0-26.0  Arm A: 5.0-26.0; Arm B: 2.0-19.5 | 1.0-26.0 | 1.0-20.5 | 1.0-19.0 | 1.0-13.0 |
| ***Section II***  **No. of treated patients** | 74 | 31 | 14 | 43 | 13 |
| **No. of progressive**  **disease patients during**  **induction phase** |  | 16 |  | 29 |  |

^1^Maintenance was calculated from the end of induction phase until discontinuation whenever it occurred first.

M: maintenance; FU: L-leucovorin plus bolus 5-fluorouracil plus 46-hour infusion of 5-fluorouracil; Folfox: FU plus oxaliplatin; Folfiri: FU plus irinotecan; Beva: Bevacizumab; Afli: Aflibercept.

One patient in arm A and one patient in arm B discontinued maintenance for reasons other than progression.

**Figure S2.** Kaplan-Meyer survival curves according to second-line chemotherapy (Arm A: folfiri/ bevacizumab; Arm B: folfiri/aflibercept) in the whole series, including 13 patients with rapidly progressing disease at I line folfox/bevacizumab.
